# Stabler Sign Secondary to Postoperative Breast Hematoma: CT Evidence of Extraperitoneal Blood Tracking

**DOI:** 10.3390/diagnostics16070981

**Published:** 2026-03-25

**Authors:** Yoen Young Chuah, Yeong Yeh Lee, Wen-Sheng Tzeng, Yow-Ling Shiue

**Affiliations:** 1 Department of Biological Sciences, National Sun Yat-Sen University, Kaohsiung 80424, Taiwan; 2 Division of Gastroenterology and Hepatology, Department of Internal Medicine, Ping Tung Christian Hospital, Pingtung 90059, Taiwan; 3 Department of Medicine, School of Medical Sciences, Universiti Sains Malaysia, Kota Bharu 16150, Malaysia; 4 Department of Radiology, Ping Tung Christian Hospital, Pingtung 90059, Taiwan; 5 Institute of Biomedical Sciences and College of Medicine, National Sun Yat-Sen University, Kaohsiung 80424, Taiwan

**Keywords:** Stabler sign, inguinal ecchymosis, breast hematoma, extraperitoneal hemorrhage, computed tomography, fascial planes, postoperative complication

## Abstract

Inguinal ecchymosis, known as Stabler sign, is classically associated with retroperitoneal hemorrhage. However, imaging confirmation of the underlying mechanism is rarely demonstrated. We report a 33-year-old woman who developed progressive right inguinal ecchymosis one week after excisional surgery for fibrocystic breast nodules. Physical examination revealed extensive bruising over the right breast and a separate ecchymotic area in the right inguinal region. Laboratory tests showed mild anemia with stable hemoglobin levels, while coagulation parameters and pancreatic enzymes were normal. Contrast-enhanced computed tomography excluded retroperitoneal or pelvic hemorrhage but revealed a large right breast hematoma. Multiplanar CT reconstructions demonstrated hyperattenuating fluid tracking along the superficial fascial planes of the anterior chest wall and abdominal wall toward the inguinal region. Anatomically, the superficial fascial system comprising Camper’s and Scarpa’s fascia provides a potential pathway for gravity-dependent migration of extraperitoneal blood. This case suggests that inguinal ecchymosis may result from extraperitoneal blood tracking from a distant postoperative hematoma rather than retroperitoneal bleeding.

**Figure 1:**
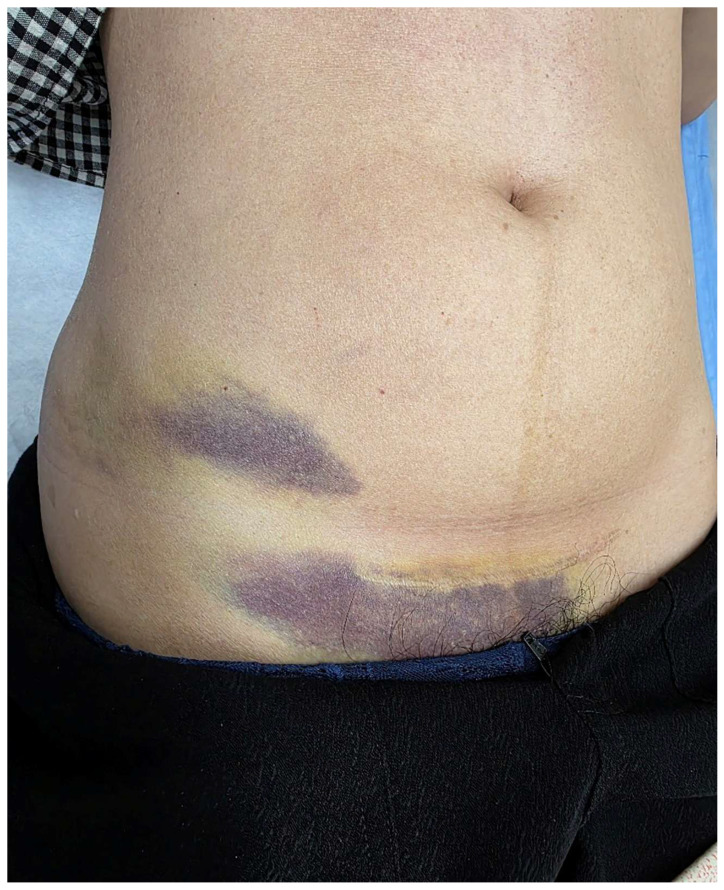
Ecchymosis over the right inguinal region, consistent with Stabler sign.

**Figure 2:**
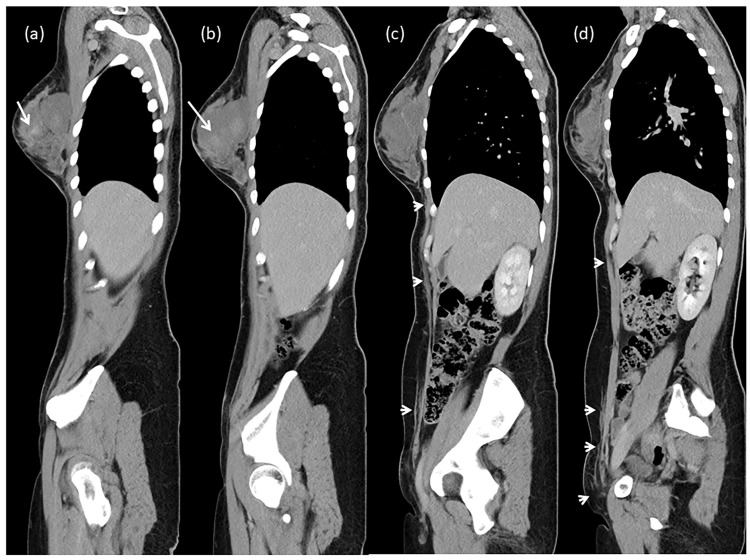
Contrast-enhanced computed tomography (CT). (**a**,**b**) Sagittal images showing a loculated hematoma in the right breast (long arrows). (**c**,**d**) Fat stranding along the right chest wall, abdominal wall, and inguinal fascial planes (short arrows), suggestive of caudal tracking of extraperitoneal blood. A 33-year-old woman presented with progressive ecchymosis over the right inguinal region for one week. She denied alcohol use, anticoagulant therapy, recent trauma, or pancreatitis. Notably, she had undergone excisional surgery for fibrocystic breast nodules three days prior to the onset of bruising. Physical examination revealed extensive ecchymosis over the right breast and a separate area of bruising over the right inguinal region, without visible continuity between the two sites (Figure 1). Laboratory investigations demonstrated normocytic anemia, with hemoglobin decreasing from 14.0 g/dL to 11.2 g/dL. Serial hemoglobin measurements remained stable thereafter. Coagulation parameters, serum amylase, and lipase levels were within normal limits. Inguinal ecchymosis developed three days postoperatively and progressively enlarged over the following week. Contrast-enhanced CT, performed seven days after symptom onset, excluded retroperitoneal hemorrhage but demonstrated a large right breast hematoma corresponding to the clinically observed breast ecchymosis. Multiplanar CT reconstructions further demonstrated hyperattenuating fluid along the superficial fascial planes of the anterior chest wall, extending inferiorly through the anterior abdominal wall toward the inguinal region. No hemorrhage was identified within the retroperitoneum, pelvis, or prevesical (Retzius) space, and no active arterial contrast extravasation was detected. These findings suggest that blood from the breast hematoma tracked along the superficial fascial system of the anterior trunk. Although not directly demonstrable, this pattern is anatomically consistent with spread along the continuous superficial fascial layers (including Camper’s and Scarpa’s fascia), which may represent a plausible explanation for the observed inguinal ecchymosis. Stabler sign, first described in 1976, refers to inguinal ecchymosis resulting from retroperitoneal hemorrhage [1]. It has been reported in association with conditions such as spontaneously ruptured neuroblastoma and portal hypertension [2,3]. Other abdominal ecchymotic signs include Cullen sign (periumbilical ecchymosis) and Grey Turner sign (flank ecchymosis). These signs are typically associated with intra-abdominal or retroperitoneal hemorrhage, including pancreatitis, ruptured pancreatic pseudocyst, traumatic bleeding, or ruptured hepatocellular carcinoma [4,5,6,7]. They are thought to arise from blood tracking along fascial planes into the subcutaneous tissues; however, this mechanism is largely inferred from clinical observation and is rarely demonstrated by imaging. The present case provides imaging findings that are compatible with hypothetical mechanism in which extraperitoneal blood originating from a postoperative breast hematoma descends along anterior fascial planes toward the inguinal region. This gravity-dependent migration may account for the delayed development of inguinal ecchymosis several days after surgery. Differential diagnoses for inguinal ecchymosis include retroperitoneal hemorrhage, femoral vessel injury, pelvic bleeding, pancreatitis-related hemorrhage, and coagulopathy. In this case, contrast-enhanced CT excluded retroperitoneal and pelvic bleeding, laboratory findings ruled out pancreatitis, and coagulation studies were normal. The absence of retroperitoneal hemorrhage, together with CT visualization of fluid tracking along the superficial fascial planes, supports a possible extraperitoneal pathway, although causality cannot be definitely established. To our knowledge, after review of the available English-language literature, CT demonstration of inguinal ecchymosis resulting from extraperitoneal blood tracking from a postoperative breast hematoma has not been previously reported. However, this report should be regarded primarily as a didactic clinical observation rather than a definitive proof of a pathophysiological mechanism. This case highlights an important diagnostic consideration: inguinal ecchymosis does not necessarily indicate retroperitoneal hemorrhage. Careful imaging evaluation is essential to identify alternative bleeding sources. Inguinal ecchymosis (Stabler sign) may reflect gravity-dependent tracking of extraperitoneal blood along fascial planes and should not be attributed to retroperitoneal hemorrhage without imaging confirmation.

## Data Availability

The original contributions presented in this study are included in the article. Further inquiries can be directed to the corresponding authors.
